# Medico-Chirurgical Society of Edinburgh

**Published:** 1888-08

**Authors:** 


### THE MEDICO-CHIRURGICAL SOCIETY OF EDINBURGH.

CLINICAL REMARKS UPON THE OPERATIVE SURGERY OF THE MALE BLADDER.

I.—*Cystotomy in Certain Cases of Enlarged Prostate with Persistent and Aggravated Irritability of the Bladder.* — Professor Thomas Annandale said: The cases referred to under this head are those in which all ordinary treatment, including careful catheterism, washing out the bladder, and general remedies fail to relieve the almost constant distressing symptoms of pain, spasm, and desire to pass urine.

Various operative procedures have from time to time been suggested for the treatment of such symptoms, all of these having the object of establishing an artificial opening communicating with the bladder, and of allowing a metal or rubber tube to be inserted, so as to drain off the urine.

Sir H. Thompson, in the *Lancet* for 1875, vol. i, suggested that in certain of these cases the bladder should be punctured above the pubes, and that a tube should be permanently retained, and he described a special instrument for the purpose. Mr. Thomas Smith, in St. Bartholomew's Hospital Reports (1881), also advocates this proceeding. Mr. Reginald Harrison, in *Lancet*, 1886, vol. i, advocates a perineal cystotomy, with the introduction of a tube in this situation. Most surgeons are, I think, now of opinion that the perineal opening is the best in the large majority of cases in which this operation is required. Such is certainly my opinion, and my reasons are, (1) that the bladder is better drained from this opening; (2) that the incision through the prostate sometimes has a beneficial effect upon the enlarged organ. Believing in the advantages of this proceeding in certain cases, my endeavor has been to improve the retained tube so as to permit of its permanent use, when required, in a form efficient and comfortable to the patient, and after several trials the apparatus now to be described appears to me to carry out these principles. An india-rubber catheter of full size having been cut short, so that its rounded end will lie in the bladder and its cut extremity project half an inch from the perineal wound, has fitted into the projecting end a short tube of hard vulcanite half an inch in length. This allows a silk or other thread with two short loops to be firmly fastened around the catheter without interfering with its canal, and by means of these loops the cathether is secured in position in the usual way. To the other end of the vulcanite tube any convenient length of india-rubber tubing can be fixed, and by placing a small stopcock on the tubing the bladder can be readily emptied at any time by turning on the tap.

In illustration of this treatment I give brief notes of the two following cases:

CASE I.—Mr. S., æt. 70, was first seen in consultation with Dr. J. Jamieson in July, 1886, on account of prostatic retention, with cystitis and great irritability of the bladder. The patient being unable to empty his bladder, regular catheterism, with washing out the bladder and general treatment, were advised. This treatment was carefully carried out by Dr. Jamieson, and I saw him occasionally in consultation. As the patient's symptoms were only temporarily removed, and as they became so much aggravated, I performed perineal cystotomy upon the 8th January, 1887. The result was most satisfactory, and a few months after the operation he was able to go about wearing the tube which I have described. Up to this date (May, 1888) he continues well, and Dr. Jamieson writes me that "from a life of misery he has passed to one of comparative comfort."

CASE II.—Mr. —, æt. 60, was seen with Dr. Dunsmure in April, 1887. His symptoms were very similar to those affecting the patient in Case I. He had been confined to bed for several months, and various forms of treatment carefully tried without giving him relief. Cystotomy was performed upon the 9th of May, 1887, and after a few weeks the tube, as described, was introduced. Since that date the patient has worn the tube and is able to go about and perform his duties.

The result obtained in these two cases is, I think, most encouraging, and it will, I hope, assist in establishing this operation as an efficient means of relief in suitable cases.

Very recently it has been suggested by Mr. McGill that a special cystotomy may be employed for the removal of a portion or portions of an enlarged prostate with a view of taking away an obstruction to the natural passage of the urine. It has been long known that projecting portions of an enlarged prostate may be safely removed. More than twenty-five years ago, when assisting the late Mr. Syme to perform lateral lithotomy upon an old gentleman, a large portion of the prostate was accidentally caught between the blades of the forceps and torn away. This patient made an excellent recovery. Similar cases have been recorded, and I think that when an exploratory incision has demonstrated a projecting enlargement of the prostate into the cavity of the bladder, which is interfering with proper micturition, the removal of this portion is a proper and justifiable proceeding.

II.—*Cystotomy in Certain Cases of Acute Retention of Urine, the Result of Enlarged Prostate.*—When acute retention of urine takes place in patients suffering from enlargement of the prostate, it may be temporarily and easily relieved, more especially if it is early and carefully treated, but the condition is not unfrequently tedious in its progress and complicated by cystitis and general irritative disturbance and fever, more particularly when treatment has been delayed or unsuccessfully employed. When the required use of the catheter irritates, or when its employment is unsatisfactory owing to the difficulty of passing it into the bladder, to the want of experience on the part of the medical attendant, or to the circumstance that the patient is distant from proper surgical aid, I am of opinion that a perineal cystotomy is the most likely means to give the patient efficient relief. No doubt in urgent cases supra-pubic aspiration or puncture is a valuable means of relieving the retention; but this proceeding can only be temporary, even if frequently repeated, and experience of it has shown that it is not without risks. On several occasions, when called to distant parts of the country to consult in regard to difficult cases of prostatic retention, I have advised and successfully performed perineal cystotomy, believing that this treatment was best for the comfort of both patient and his medical attendant.

III. — *Cystotomy for the Removal of Tumors of the Bladder.*—The experience of this operation has already proved that in favorable cases—that is, in cases of tumors having little or no malignant tendencies — encouraging results are obtained. It cannot yet be said that the exact diagnosis of the presence of a tumor in the bladder can always be made, nor can the exact nature of the tumor, if present, be always determined by mere symptoms, microscopic examination of the urine, or external examination, but an exploratory incision will in all cases of doubt decide the question.

From my experience in connection with operations for the removal of bladder tumors I would wish to express an opinion as to the best form of cystotomy in such cases. I have no hesitation in saying that tumors of the bladder can be most accurately and thoroughly removed by making both a supra-pubic and perineal incision. If the forceps or other extracting instrument is introduced through the perineal wound and their blades, guided by one or more fingers, passed into the bladder through the supra-pubic wound, an accurate removal of the growth is, in my opinion, most thoroughly obtained, more particularly in the case of broad-based tumors. It is quite possible to remove a tumor of the bladder through either a perineal or supra-pubic opening, but I believe that by means of the two openings a greater accuracy of removal is possible.

My usual practice is to first make a supra-pubic cystotomy, and then a central perineal one, in all cases in which the diagnosis of the presence of a removable tumor is well determined; but when the diagnosis is doubtful, I first make a central perineal exploratory incision, and then, if required, follow this up by a supra-pubic cystotomy.

IV.—*Cystotomy for Persistent Irritability of the Bladder Unrelieved by Ordinary Careful Treatment.*—Cases of this nature are not uncommon, and they may or may not be associated with symptoms of cystitis. When all ordinary treatment fails to relieve, perineal cystotomy gives, in my opinion, the best chance of curing or relieving the condition, and it is from my experience much more effective than any drainage of the bladder by the retention of a catheter or tube passed along the urethra. In some of these cases little or no change in the bladder structures can be detected; but in others I have found superficial ulceration or a velvety condition of the mucous membrane. Needless to say that before practicing cystotomy in any case of this kind all sources which are known to produce symptoms of irritability of the bladder should be carefully investigated.

*Note.*—The recent suggestion that the endoscope may be employed by passing it through a perineal wound, such as is made in perineal cystotomy, is, in my opinion, a most valuable one, and is likely to much assist the correct diagnosis of obscure bladder conditions.

### EMPYÆMA OF THE SUPERIOR MAXILLARY ANTRUM WITH ONLY NASAL SYMPTOMS.

P. M'Bride, M. D., F. R. C. P. Ed., F. R. S. E., Surgeon to the Ear and Throat Department, Royal Infirmary, and Lecturer on Diseases of the Ear and Throat, Edinburgh School of Medicine, read the following paper:

Among both surgeons and specialists there has been, and I may even say there still is, a tendency to regard suppuration of the lining membrane of the maxillary antrum as invariably attended with pain, swelling, and distention of the osseous walls of the cavity. The condition to which I propose now to direct attention is associated with none of these symptoms, and the patients usually come to the surgeon complaining only of a constant or periodic discharge of pus from the nose. Sometimes the secretion is offensive, and then both its smell and taste may be perceived by the sufferer; in other instances, however, it is free from fœtor. Inasmuch as one antrum only is commonly affected, the discharge is usually found to come from one nostril—although it must not be forgotten that as the two nostrils communicate posteriorly any large quantity of secretion finding its way into one may in part escape by the other, more especially while blowing the nose. I have laid some stress upon this point because the unilateral character of the discharge is often only discovered after careful questioning.

Very often, too, the patient has never observed that the quantity of discharge is altered by changing the position of the head, although I believe that a careful rhinoscopic examination will commonly prove that this phenomenon is present. In most instances inquiry will elicit a history of faceache, sometimes extremely slight, or at least of a period of discomfort in the region of the cheek; but this is frequently only discovered by strict questioning, because to the patients there seems to be no connection between the two conditions.

For the most part there is neither pain nor discomfort when the case is examined, because the suppuration has become chronic, and the pus discharges freely through the natural orifice (or orifices) into the nose. Fraenkel,* however, states that in not a few cases frontal headache is complained of, and I have seen the same

*"Berlin Klin. Wochens.," No. 16, 1887.

symptom in one of my own cases. I was at one time inclined to regard this symptom as a reflex neuralgia, but am now more disposed to adopt the explanation suggested by Killian.* It will be remembered that the normal opening of the antrum into the nose is close to that of the frontal sinus. We shall presently—when discussing the objective signs—see that the nostril corresponding to the affected side usually shows swelling of the mucous membrane. As a sequence of this tumefaction closure of the communication between the frontal sinus and the nose occurs with the natural result, that as the contained air is absorbed, the frontal cavity is placed under abmormal physical conditions. It is even conceivable that in this way secondary inflammatory changes and exudation might result in the frontal sinus. The possibility of forehead pain and tenderness being due to antral disease is a matter of great importance, as its presence might easily lead to the diagnosis of suppuration in the frontal or ethmoidal cells.

No doubt the class of cases we are considering have been long since recognized by individual surgeons, and probably also by dentists. This may be gathered by a careful perusal of such authors as Erichsen,† Heath,‡ Lefferts,§ Scech,‖ Salter,¶ and others.

Neither in the works just referred to, however, nor in others, have I found this subject treated of with the care and consideration which it merits. Thus, Moldenhauer,** who has written the most recent and one of the best text-books on the nose, describes under the designation "Disease of the Osseous Portion of the Nose," a class of cases in which, despite his reference to Ziem's paper, he seems to assume that the nasal discharge is due to limited caries. Of the six cases there referred to, I believe, from the author's observations, that in every one the symptoms must have been due to chronic suppuration of the lining membrane of the antrum of Highmore. I have stated these facts to show that until quite recently, suppuration of the antrum without retention of pus was in many cases overlooked. To Ziem,* therefore, we must give the credit of first directing the attention of specialists to the important fact that a discharge of pus from the nose is often due to suppuration within the antrum. It seems to me, however, that we cannot hold this authority guiltless of extreme zeal for the neglected cause which he espoused. Yet, notwithstanding this, he was able to show a large number of successful results, although he seems to have opened the antrum in almost every case of purulent discharge from the nose for which he was consulted. The most scientific contribution, however, on this question is from the pen of B. Fraenkel.† As he very properly points out, the pus in these cases can be seen to issue from the region of the hiatus semilunaris. If then the nose be thoroughly cleansed, and the secretion be seen soon afterwards to appear in the middle meatus, we may suspect antral mischief but, as Fraenkel remarks, "Not all pus that is poured into the middle meatus through the hiatus comes from the antrum." For it may also proceed from the frontal sinus. In order to differentiate the two conditions, Fraenkel carefully cleanses the nostril and makes the patient sit with his head down, a position which would render drainage from the frontal sinus into the nose impossible. If, after this, more pus be found, he concludes that the maxillary sinus is at fault.

Let us now take a final glance at the clinical feature of the affection we are dis-

*"Monatsschrift für Ohrenheilkunde," etc., Oct. and Nov. 1887.

†"Science and Art of Surgery," 1887, vol. ii., p. 474.

‡"International Encyc. of Surgery" (Ashhurst), vol. v.

§ Ibid,

‖ "Diseases of the Mouth, Nose, and Throat." Translated by Blaikie, 1886.

¶"Dental Pathology and Surgery," 1874.

**"Krankeiten der Nasenhöhlen," etc., 1886.

*"Monatsschrift für Ohrenheilkunde" (Feb., March, April, 1886).

†"Berlin Klin. Wochens.," 16, 1888.

cussing. The patient complains of a unilateral discharge of pus, which sometimes trickles from the nose in a continuous stream, and may be fœtid or sweet; in some persons the discharge seems to occur periodically. Careful examination of the affected nostril shows that the secretion comes from a point just below the anterior extremity of the middle turbinated body; sometimes, however, it is sufficiently copious to cover the posterior surface of the palate, posterior end of the inferior turbinated body, etc. If the nostrils be syringed out, and the drop of pus wiped away from the middle meatus, and the head thrown forward with the forehead down, the secretion is seen, on examination undertaken immediately afterwards, to be increased in quantity. Anterior rhinoscopy often shows the mucous covering of the inferior and middle turbinated bodies to be hypertrophied, or at all events in a state of erectile swelling. This last named condition can, of course, be temporarily overcome by the application of cocaine or menthol—a proceeding which is often necessary to obtain a better view of the parts.

Posterior rhinoscopy is merely useful in excluding other conditions. The diagnosis of the form of empyæma of the antrum under discussion is arrived at by a process of exclusion. The frontal sinus is eliminated by Fraenkel's method, and besides this cavity is rarely affected by itself. This, Zuckerkandl,* whose work on the normal and pathological anatomy of the nose is probably the most complete in existence, has never met with isolated inflammation of the frontal sinus. A careful rhinoscopic examination will exclude all other possible causes of unilateral nasal discharge, *e. g.*, the presence of a foreign body or rhinolith, ulceration, inflammation of the bursa pharyngea, ozæna, caries, etc.

There is, however, one condition which deserves more than passing notice, and which was first pointed out to me by Dr. Cotterill, in a case where he operated at my suggestion, viz., a marked redness of the gum corresponding to the affected antrum. This was found in the last case but one which I have examined; in the last the same phenomenon was present. I am not aware that this condition has been so far observed by the few authors who have written on empyæma of the antrum without the usually described symptoms. As to the cause of this form of antral affection, I think by far the most common is to be sought in the presence of decayed upper teeth, usually the bicuspids or first molar. I have met with one case, however, in which the antral inflammation was set up by either long continued nasal catarrh, or by douches used for its cure.

In conclusion, I shall briefly record two cases which seem to me fairly typical.

Mr. C. consulted me in August, 1887, and has had a discharge of matter from the left nostril for five years or so, and all remedies so far recommended having proved useless.

*Objective Examination—Post-Rhinoscopy.*—Parts fairly healthy, except some thickening of posterior extremities of the inferior turbinated bodies, especially on left side.

*Anterior Rhinoscopy.*—Right nostril almost normal; left, pus coming from anterior part of middle meatus, increased by holding head over to the right. No pain or swelling over antrum, but has had a gumboil repeatedly over the stump of the second bicuspid, where there is now a sinus. The tooth was extracted on my recommendation by a dentist (Mr. Watson), and the antrum opened through the socket, a proceeding which gave escape to a considerable quantity of pus. Systematic irrigation, carried out by the patient, freed him from his troublesome and distressing affection.

Mr. M. consulted me 18th December, 1887. For nine years patient has used a nasal douche as part of his toilet; this was ordered on account of an ear affec-

* "Normal und Path. Anat. der Nasenhöhle," etc., p. 168.

tion at that time; occasionally during its employment a little pellet came away. He now, since July, complains of fœtid discharge from the right nostril. There was no history of pain in the antrum; but once during a railway journey he felt a curious drawn feeling in his face. He thought little of this, and it immediately passed off. In this connection it may be stated that the patient is extremely neurotic and would probably feel pain acutely.

*Nasal Condition.* — Slight tenderness over right cheek on firm pressure; teeth quite perfect; discharge increased by bending head over to left and also keeping forehead down.

*Post-Nares.*—Some purulent discharge; no ulcer or other condition to account for its presence.

*Anterior Nares.*—Left nostril some catarrh; no pus. Right nostril, inferior turbinated body in a state of erectile swelling, which subsides under cocaine; pus coming from middle meatus, and showing pulsation.

*Nostrils.*—Blown separately, right yields copious fœtid purulent discharge; from the left there comes only serous sweet discharge. No tenderness of the teeth; congestion of the upper gum and the right of the middle line; no bulging anywhere.

On the 22d December the patient was again examined, in conjunction with Mr. Duncan, and on this occasion the second bicuspid and first molar on the affected side were found to be loosened, whereas four days previously they were quite normal. Mr. Duncan after this opened the antrum through the alveolar process, and let out a quantity of fœtid pus. The case is still under treatment, but under continued irrigation, with boracic lotion and free drainage, is sure to do well. I have detailed this case at length, in order to illustrate the fact that the absence of bad teeth is no guarantee against antral mischief. As I have before said, antral empyæma is commonly unilateral. To make this paper complete, however, it is necessary to state that in a few cases Ziem found the affection to exist on both sides. So far I have not met with such a case, but the diagnosis could, no doubt, be arrived at without difficulty.

In conclusion, it remains to refer briefly to the treatment. I do not think that Stoerk's suggestion to treat these cases by the introduction of a tube into, or, if this be impossible, up to the natural opening between the antrum and the nose, and thus to irrigate the cavity, is at all advantageous. Manifestly, this line of treatment, even if in the end successful, must entail so much time and trouble as to weary the average patient. No doubt the establishment of a counter-opening is more scientific and satisfactory. Such a counter-opening may be made through the socket of a tooth, through the alveolar process, or finally through the outer wall of the nose near its floor, as suggested recently by Mikulicz. After a free drain has been established, irrigation of the antrum must be undertaken, and continued until the case is cured. For injection, such fluids as boracic lotion, or water containing small quantities of iodine or chloride of zinc, may be used, according to the indications afforded by each particular case. Whether the insufflation of powders in addition (boracic acid, iodoform. etc.) would prove more rapidly effectual, I am not yet prepared to say.

### DISCUSSION.

The PRESIDENT said he could corroborate much of what had been communicated by Dr. M'Bride. In many of these cases there was no pain, and the patient had no suspicion of antral disease. Sometimes the frontal, ethmoidal, and the sphenoidal sinuses might contribute as a means of keeping up the nasal discharge, but none of the sinuses were subject to the same irritation or liability to morbid conditions as the antrum. He believed that when carious or loose, or in any way suspicious teeth existed, with symptoms of an ozænic tendency, they should be extracted. The antrum retained its secretion much more than the other sinuses. Its floor was really lower than the palate, and was deep behind the posterior bicuspid, while the opening into the meatus was near its roof. In this way he had no doubt that in these antral cases, by its retention the mucus or muco-pus became very offensive. When these cases required opening, he thought it was best done above the external alveolar process. Such an opening closed much easier than one made through the socket of a tooth which often healed with difficulty, a sinus or fistulous opening continuing long after it was necessary. Opening the antrum was not required in every case. Where no obstruction or distention existed, he had seen removal of the offending teeth followed by disappearance of all the symptoms. In some jaws the antrum was not easily opened from the outside.

Professor ANNANDALE considered that the most important and practical pointof Dr. M'Bride's paper was that such cases were often not discovered. He was quite willing to admit that improved rhinoscopic examinations might be an assistance in their diagnosis, but he was inclined to think, from his surgical experience, that there were always local symptoms which pointed to the presence of pus in the antral cavity. His experience was that if there was a discharge from one nasal cavity, the antrum on that side should be carefully examined, and invariably he had found that a practical surgeon accustomed to examine carefully would be able to find some tenderness or slight enlargement, showing something wrong with the antrum. The last case he saw was that of a young gentleman, who had undergone a variety of treatment for a nasal dischage without curative result. He found slight tenderness, and opened into the antrum, and was rewarded by finding pus. He was strongly of opinion that the best treatment was to open into the cavity above the alveolar margin. If there were diseased or loose teeth, the fangs of which were causing irritation, it was well to remove them, but his experience was that the opening above gave better and quicker results as regards cure. He found a considerable difference in regard to the size and even the position of the antrum. In some cases he had had to puncture very near to the floor of the orbit to effect an opening.

Dr. HORSLEY asked if Dr. M'Bride had any experience as to which of the various openings recommended, through the socket of a tooth, the external alveolar process, or through the nose, as practiced by the German surgeons, gave the best result?

Dr. COTTERILL believed that the best plan was to attack the condition from the outside, the alveolar, not the nasal surface. The opening into the antrum would be on too high a level if made from the inside. Some cases treated by the internal opening were reported as cures in some of the medical journals a short time ago, but without sufficient details to enable one to judge. In the last case he had operated on, there was no enlargement and only slight tenderness on pressure. He made the opening through the canine fossa. The teeth had been removed some time before, and the sockets filled up, and an opening through them would have been more difficult He had the cavity syringed out twice a day through a glass drainage-tube covered with rubber at the end. This was done for a fortnight till pus ceased to appear. Five days after removal of the tube the cavity had filled up again. It was reopened, the tube reinserted, and left in for about three weeks with a successful result.

Dr. AITKEN mentioned a case in which there was no difficulty about the diagnosis. The pus had made its way through the hard palate, and gave rise to two swellings in the roof of the mouth. Behind these swellings was a small ivory exostosis. The first attempt to open into the cavity was made by an endeavor to extract a stump, thought to be the canine, but owing to the pain, and to the fact that the antral wall was so thin, the antrum was opened above the alveolar margin. Immediately that was done the stump dropped out, showing the considerable part the atmospheric pressure plays in maintaining the teeth in their sockets. A drainage tube was kept in for about ten days, and the cavity was washed out with an antiseptic solution introduced by means of a Eustachian catheter attached to a syringe. The case was mentioned to show the great importance of free drainage and perfect asepticism.

Dr. M'Bride thought Professor Annandale was inclined to criticise the value of the rhinoscope in those cases on the ground that the experienced surgeon would find external evidence of the presence of pus in the antrum. But he had shown that in them there need be no bulging, no swelling, no pain, no tension, though there might be some congestion of the gum, and in all but one of his cases there had been diseased teeth. Unilateral discharge was not sufficient evidence of antral affection, inasmuch as it might be due to syphilis, rhinoliths, foreign bodies, or other causes. He thought that in many cases the arch of the palate might be taken as a guide to the size and position of the antrum.

The President said the antrum varied very much in its capacity, shape, and position in different subjects and at different periods of life. Where the arch of the palate was high it was very much more out of reach. It could not well be opened through the socket of the canine tooth, as its limit anteriorly was about the second bicuspid. It did not seem, however, to be what might be called antral teeth alone that were connected with this affection, as removal of diseased incisors had been of marked benefit in some cases, probably by the removal of diffuse irritation throughout the superior maxilla.

Mr. A. G. Miller said he had once suffered from an acute abscess of the right antrum. He got great relief from the use of warm fomentations, and found that when he lay on his left side he was relieved of pain, and the matter trickled away slowly and intermittently. He cured himself by lying in bed in this position for two or three days. He thought that something of the kind might be tried in chronic cases along with antiseptic irrigation of the nares.—*Edinburgh Medical Journal.*

